# Tonality Tunes the Statistical Characteristics in Music: Computational Approaches on Statistical Learning

**DOI:** 10.3389/fncom.2019.00070

**Published:** 2019-10-02

**Authors:** Tatsuya Daikoku

**Affiliations:** Department of Neuropsychology, Max Planck Institute for Human Cognitive and Brain Sciences, Leipzig, Germany

**Keywords:** creativity, Markov model, n-gram, information theory, corpus, prediction, composition, implicit learning

## Abstract

Statistical learning is a learning mechanism based on transition probability in sequences such as music and language. Recent computational and neurophysiological studies suggest that the statistical learning contributes to production, action, and musical creativity as well as prediction and perception. The present study investigated how statistical structure interacts with tonalities in music based on various-order statistical models. To verify this in all 24 major and minor keys, the transition probabilities of the sequences containing the highest pitches in Bach's Well-Tempered Clavier, which is a collection of two series (No. 1 and No. 2) of preludes and fugues in all of the 24 major and minor keys, were calculated based on *n*th-order Markov models. The transition probabilities of each sequence were compared among tonalities (major and minor), two series (No. 1 and No. 2), and music types (prelude and fugue). The differences in statistical characteristics between major and minor keys were detected in lower- but not higher-order models. The results also showed that statistical knowledge in music might be modulated by tonalities and composition periods. Furthermore, the principal component analysis detected the shared components of related keys, suggesting that the tonalities modulate statistical characteristics in music. The present study may suggest that there are at least two types of statistical knowledge in music that are interdependent on and independent of tonality, respectively.

## Introduction

### Prediction and Production in the Statistical Learning

The brain is innately equipped with statistical learning (SL) machineries that model external phenomena as a dynamical system that encode the probability distributions. The SL is thought as an implicit process in which the brain automatically calculate transitional-probability (TP) distribution of sequential information such as music and language (Saffran et al., 1996; Cleeremans et al., 1998). Furthermore, based on the internalized statistical model, it can predict a future state and optimize action for achieving a given goal (Monroy et al., 2017a,c) to resolve the uncertainty of information (Friston, 2010). The SL has also be thought to contribute to the encoding of the complexity in the information (Hasson, 2017), and to acquisition of musical and linguistic knowledge including tonality (Daikoku et al., 2016) and syntax (Daikoku et al., 2017a). For example, an increasing volume of literature also demonstrates that SL and the knowledge associate with human's action (Zubicaray et al., 2013; Monroy et al., 2017a,b, 2018) and decision-making (Schwartenbeck et al., 2013; Friston et al., 2014, 2015; Pezzulo et al., 2015). For example, motor cortex activity contributes to SL of action words (Zubicaray et al., 2013). Furthermore, cerebellum and cerebral cortex partially share same network responsible for the interalized statistical model. That is, statistical knowledge formed in cerebral cortex may be sent to the cerebellum that is thought to play important roles in prediction of sequences (Lesage et al., 2012; Moberget et al., 2014), motor skill learning (Ito, 2008), habit learning (Friston et al., 2016), generalization or abstraction based on transitional probabilities (Shimizu et al., 2017), efficient performance in a learned context (Balsters et al., 2014). These findings may suggest that the internalized statistical model affects production of music (i.e., composition) (Daikoku, 2019a), the creativity (Wiggins, 2018), and individuality of artistic expression (Daikoku, 2018b) as well as the prediction and perception (Daikoku, 2019c). It is, however, unknown how the acquired statistical knowledge influences the production of music.

### Statistical Learning Machinery in Musician

According to recent studies, musicians are better statistical learners than non-musicians (Francois and Schön, 2011; François et al., 2012; Hansen and Pearce, 2014; Przysinda et al., 2017; Elmer and Lutz, 2018). Furthermore, it is suggested that, through long-term musical training, musicians optimize the brain's probabilistic model of SL, and that the musically-optimized SL model allow the brain to precisely and efficiently predict tones during SL of another musical and auditory sequences (Francois and Schön, 2011; Kim et al., 2011; Hansen and Pearce, 2014; Przysinda et al., 2017). Recent computational studies also suggested that, from early to late periods in the composer's lifetime, the transitional probabilities of familiar phrase in each piece of music were gradually decreased (Daikoku, 2018d, 2019a). These findings were prominent in higher-, rather than lower-order SL models. These studies suggest that the higher-, rather than lower-, order statistical knowledge (Daikoku, 2018a) may be susceptible to long-term experience that modulates brain's SL model (Hansen and Pearce, 2014). Furthermore, computational studies on improvisation music suggested that lower-order SL models represented general characteristics shared among musicians, whereas higher-order SL models detected specific characteristics unique to each musician (Daikoku, 2018b). In this context, it can be hypothesized that statistical models in music, which may reflect the composer's statistical knowledge, interact with the music-specific structures of tonality. To our knowledge, however, few studies have examined how TP in music interacts with the tonalities. To understand the characteristics of music from interdisciplinary aspects that include informatics, musicology, and psychology, it is important to verify the interaction between tonality and statistical structure in music, especially regarding strategies of musical composition.

### Computational Modeling

The computational model and simulation have been used to understand SL systems (e.g., Pearce and Wiggins, 2012; Rohrmeier and Rebuschat, 2012; Daikoku, 2018a, 2019b; Wiggins, 2018). Particularly, the prediction and production of SL is partially supported by chunking hypothesis that learning is based on extracting, storing, and combining small chunks. For example, information-theoretical models including *Markovian* processes have been applied to neurophysiological studies of SL in human brain as well as computational simulation (Pearce et al., 2010; Pearce and Wiggins, 2012; Daikoku et al., 2014, 2015, 2017b, 2018; Yumoto and Daikoku, 2016, 2018; Daikoku and Yumoto, 2017, 2019; Daikoku, 2018c). These neurophysiological experiments showed consistent evidence: neural activities for stimuli with high information content (i.e., low probability) are larger than those with low information content (i.e., high probability). This neural phenomenon is in agreement with a *Bayesian* hypothesis in theoretical neurobiology that the brain encodes probabilities (beliefs) about the causes of sensory data, and that these beliefs are updated in response to new sensory evidence based on Bayesian inference (Kersten et al., 2004; Knill and Pouget, 2004; Doya et al., 2007; Friston, 2010; O'Reilly et al., 2012; Parr and Friston, 2018; Parr et al., 2018). That is, information-theoretical computational models including *Markovian* processes can capture a variety of neurophysiological phenomena on prediction, chunk formation, action, and production in the framework of SL theory.

### The Aim of the Present Study

**Figure 1:**
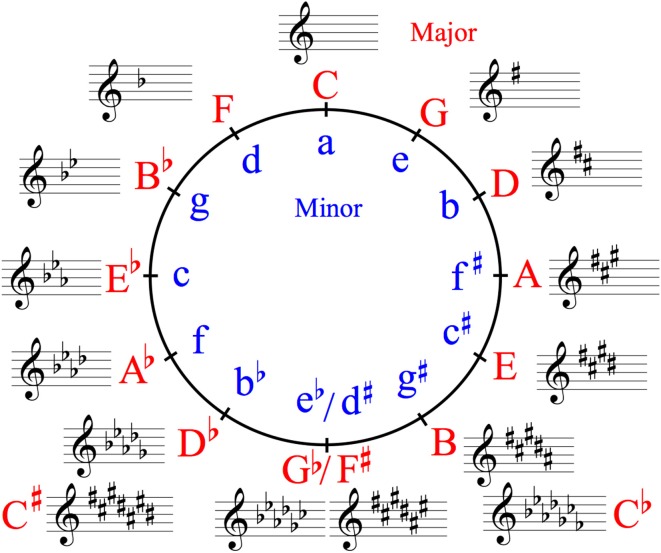
Circle of fifths showing all 24 major and minor keys in Western classical music. A related key is one sharing many common tones with an original key, as opposed to a distant key. In music, such a key shares all, or all except one, pitches with a key with which it is being compared, and it is adjacent to it on the circle of fifths and its relative majors or minors. In a related key, a subdominant key has one more flat around the circle of fifths, and a relative key has the same key signature.

This study aimed to examine how the statistical structure interacts with tonality. To verify the statistical relationships in all the keys of Western classical music (Figure 1), the TPs of the sequences containing the highest pitches in Bach's Well-Tempered Clavier, BWV 846–893, which is a collection of two series (No. 1 and No. 2) of preludes and fugues in all of the 24 major and minor keys (Figure 1), were calculated using six different orders of Markov or n-gram models (i.e., first- to sixth-order Markov chains). Johann Sebastian Bach (1685–1750) was a composer during the Baroque period, who contributed to the development of musical tonality and the Western classical music theory (Rohrmeier and Cross, 2008). His music is often used to verify the probabilities of musical sequences (Rohrmeier and Cross, 2008; Kim et al., 2011). Particularly, to understand the relationships between tonality and statistical structure in music, the Well-Tempered Clavier may be one of the best mediums because it is a collection of music containing all 24 of the major and minor keys by a single composer in Western classical music. Thus, the statistics in each piece of music with a key in the Well-Tempered Clavier could be, in part, regarded as an approximation of the statistics of the entire range of Western classical music in each key. Thus, to extract statistical knowledge dependent on keys and tonalities, the present study verified the statistical structure in each key and tonality. The TPs of each sequence were compared among tonalities (major and minor), two series (No. 1 and No. 2), and music types (prelude and fugue). It was hypothesized that the statistical structure in music interacts with the tonality in music. If so, these findings suggest that music-specific knowledge of tonality modulates statistical knowledge in music.

## Methods

**Figure 2:**
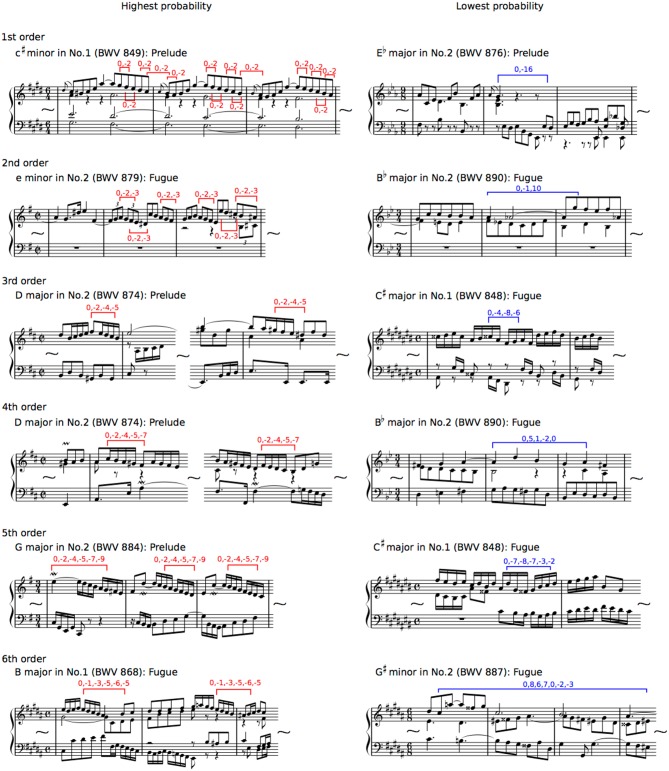
Representative phrases of sequential patterns with mean highest (left and red) and lowest (right and blue) probabilities in the six different hierarchical models of TPs for the Well-Tempered Clavier, BWV 846–893, which is a collection of two series (No. 1 and No. 2) of Preludes and Fugues in all 24 major and minor keys that was composed for solo keyboard by Johann Sebastian Bach.

The Well-Tempered Clavier, BWV 846–893, which is a collection of two series (No. 1 and No. 2) of Preludes and Fugues in all 24 major and minor keys that was composed for solo keyboard by Johann Sebastian Bach, was used in the present study. Electronic scoring data of highest pitch were extracted from the Extensible Markup Language (XML) files. The highest pitches were chosen based on the following definitions (Figure 2): the grace notes were excluded, the pitches with slurs can be counted as one, and the highest pitches that can be played at a given point in time. According to SL theory, the brain automatically computes *n*th-order TPs of sequence. The transitional probability is a conditional probability of an event B given that the latest event A has occurred, written as P(B|A). The first- to six-order TPs of an event in SL were calculated from conditional probability (P) of an event e_n+1_, given the preceding n events, based on the first- to six-order Markov models (*n* = 1–6):

$$\begin{array}{l} P\left(e_{n+1}|e_{n}\right)\;=\;\frac{P\left(e_{n+1}\cap e_{n}\right)}{P\left(e_{n}\right)} \end{array}$$

From the perspective of psychology, the formula can be interpreted as positing that the brain predicts a subsequent event e_n+1_ based on the preceding events e_n_ in a sequence (for more details, see Daikoku, 2018c). In other words, learners expect the events with higher TPs based on the latest n states (i.e., *n*th-order), whereas they are likely to be surprised by events with lower TPs. Then, all of the pitch transitions were numbered so that the first pitch was 0 in each sequential pattern, and an increase or decrease in a semitone was 1 and −1 based on the first pitch, respectively (Figure 2). This reveals interval patterns but not pitch pattern, and eliminates the effects of the change of key on sequential patterns. This procedure was employed because the interpretation of the change of key depends on musicians, and it is difficult to define it in an objective manner. Thus, the results in this study may represent statistics based on relative, rather than absolute pitches. To verify the difference in statistical structures between prelude and fugue, the sequential patterns that appear in all pieces of music that were divided between prelude and fugue were only used in the present study (1st: 4). In the second- to sixth-order Markov chains, sequential patterns that appear in all music could not be detected. The empirical logit transformation was applied to normalize the TPs. The empirical logit transform allows data distribution to be normalized, and is used for a tolerence such that infinity is not returned when the argument is zero (0%) or one (100%). Thus, it is applicable when the TP values, which often show 0% and 100%, are analyzed. Then, we conducted repeated-measure analysis of variances (ANOVAs) based on a factor type (prelude vs. fugue), a factor tonality (major vs. minor), a factor number (No. 1 vs. No. 2), and a factor sequence (4 sequences) for the 1st-order Markov model. Bonferroni-corrected *post-hoc* tests were conducted for further analysis (Statistical significance levels: *p* < 0.05). It has been suggested that the TP distribution represents statistical characteristics in music (Daikoku, 2018b). Thus, using the nth-order TP distributions, the musical characteristic in each tonality was verified by correlation analysis. Furthermore, based on the result of correlation analysis, the TPs, in which there are a number of correlations of at least 0.3 (30), were analyzed by principal component analysis (PCA). The criteria of eigenvalue were set over 1. The first three components (i.e., the first to third highest cumulative contribution ratios) were adopted in the present study. The present study focus on the values of “*loadings*.” The loading has generally been understood as the weights for each original variable when calculating the principal component. The representative phrases of sequential patterns with mean highest and lowest probabilities were decoded as musical scores (Figure 2). The criterion of the eigenvalue was set over 1 (Statistical significance levels: *p* < 0.05).

## Results

### ANOVA

**Figure 3:**
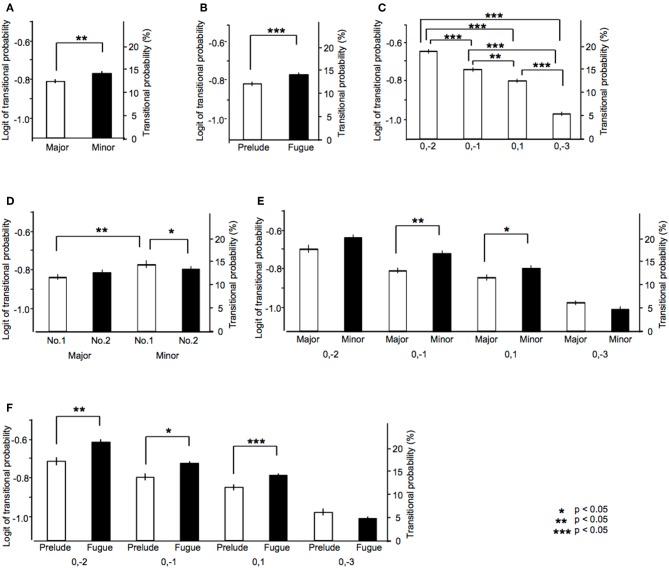
The results of ANOVA in analysis 2. The main effects of **(A)** tonality, **(B)** type, and **(C)** sequence. The interactions of **(D)** tonality-number, **(E)** tonality-sequence, and **(F)** type-sequence.

Higher-order of model represents exponentially larger numbers of sequential patterns: over forty in the first-order models, 600 in the second-order models, 3,500 in the third-order models, 9,000 in the fourth-order models, 15,000 in the fifth-order models, 20,000 in the sixth-order models. The results were shown in Figure 3. The main tonality effect showed that TPs of sequence that appear in all music in major key were lower than those in minor key [*F*_(1, 11)_ = 9.83, *p* = 0.009, partial η^2^ = 0.47; Figure 3A]. The main type effect showed that TPs of sequence that appear in all music in preludes were lower than those in fugues [*F*_(1, 11)_ = 140.74, *p* < 0.001, partial η^2^ = 0.93; Figure 3B]. The main sequence effect were significant [*F*_(2.16, 23.76)_ = 26.54, *p* < 0.001, partial η^2^ = 0.71; Figure 3C]. The TPs of [0, −2] was significantly higher compared with those of [0, −1], [0, 1], and [0, −3] (all: *p* < 0.001). The TPs of [0, −1] was higher compared with those of [0, 1] (*p* = 0.005) and [0, −3] (*p* < 0.001). The TPs of [0, 1] was higher compared with those of [0, −3] (*p* < 0.001). The tonality-number interactions were significant [*F*_(1, 11)_ = 7.57, *p* = 0.019, partialη^2^ = 0.41; Figure 3D]. In No. 1 of a collection of two series, the TPs in major key were significantly lower than those in minor key (*p* = 0.001). In minor key, the TPs in No. 1 were higher compared with those in No. 2 (*p* = 0.044). The tonality-sequence interactions were significant [*F*_(1.68, 18.46)_ = 5.35, *p* = 0.019, partial η^2^ = 0.33; Figure 3E]. In sequences of [0, −1], the TPs in major key were significantly lower than those in minor key (*p* = 0.001). In sequences of [0, 1], the TPs in major key were significantly lower than those in minor key (*p* = 0.019). In major key, the TPs of [0, −2] was higher compared with those of [0, −1], [0, 1], and [0, −3] (all: *p* < 0.001). The TPs of [0, −1] was higher compared with those of [0, −3] (*p* < 0.001). The TPs of [0, 1] was higher compared with those of [0, −3] (*p* = 0.002). In minor key, the TPs of [0, −2] was higher compared with those of [0,−1] (p = 0.001), [0, 1] (*p* < 0.001), and [0, −3] (*p* < 0.001). The TPs of [0, −1] was higher compared with those of [0, 1] (*p* = 0.006) and [0, −3] (*p* < 0.001). The TPs of [0, 1] was higher compared with those of [0, −3] (*p* < 0.001).

The type-sequence interactions were significant [*F*_(1.85, 20.34)_ = 7.64, *p* = 0.004, partial η^2^ = 0.41]. In sequences of [0, −2], the TPs in prelude were significantly lower than those in fugue (*p* < 0.001). In sequences of [0, −1], the TPs in prelude were significantly lower than those in fugue (*p* = 0.012). In sequences of [0, 1], the TPs in prelude were significantly lower than those in fugue (*p* = 0.005). In prelude, the TPs of [0, −2] was higher compared with those of [0, −1], [0, 1], and [0, −3] (all: *p* < 0.001). In fugue, the TPs of [0, −2] was higher compared with those of [0, −1], [0, 1], and [0, −3] (all: *p* < 0.001). The TPs of [0, −1] was higher compared with those of [0, 1] (*p* = 0.006) and [0, −3] (*p* = 0.001). The TPs of [0, 1] was higher compared with those of [0, −3] (*p* = 0.010). In fugue, the TPs of [0, −2] was higher compared with those of [0, −1], [0, 1], and [0, −3] (all: *p* < 0.001). The TPs of [0, −1] was higher compared with those of [0, 1] (*p* = 0.047) and [0, −3] (*p* < 0.001). The TPs of [0, 1] was higher compared with those of [0, −3] (*p* < 0.001).

### Correlation Analysis

**Figure 4:**
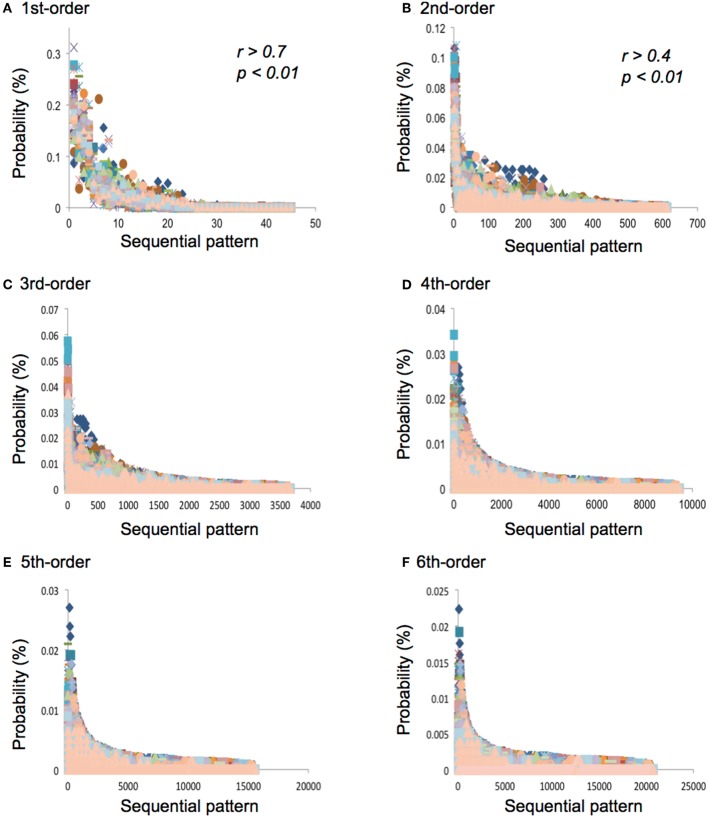
**(A)** The first-, **(B)** second-, **(C)** third-, **(D)** fourth-, **(E)** fifth-, and **(F)** sixth-order TPs in each sequential pattern. The horizontal and vertical axes represent sequential patterns and the TPs, respectively. The sequential patterns were arranged in descending order in each hierarchy.

All the results of the correlation analysis are shown in Supplementary Material. In the first-order TPs, all the pieces of music are strongly (0.7 ≦ |r| < 1.0, *p* < 0.01; Supplementary Material, red) or moderately (0.4 ≦ |r| < 0.7, *p* < 0.01; Supplementary Material, green) related to each other (Figure 4A). In the second-order TPs, all the pieces of music are moderately (0.4 ≦ |r| < 0.7, *p* < 0.01; Supplementary Material, green) or weakly (0.2 ≦ |r| < 0.4, *p* < 0.01; Supplementary Material, yellow) related to each other (Figure 4B). In the third- and fourth-order TPs, some of the music is weakly (0.2 ≦ |r| < 0.4, *p* < 0.01; Supplementary Material, yellow) related to each other (Figures 4C,D). There are more weak correlations in the third-order than in the fourth-order TPs. In the fifth- and sixth-order TPs, no strong, moderate, or weak correlations were detected (Figures 4E,F).

### Principal Component Analysis

**Figure 5:**
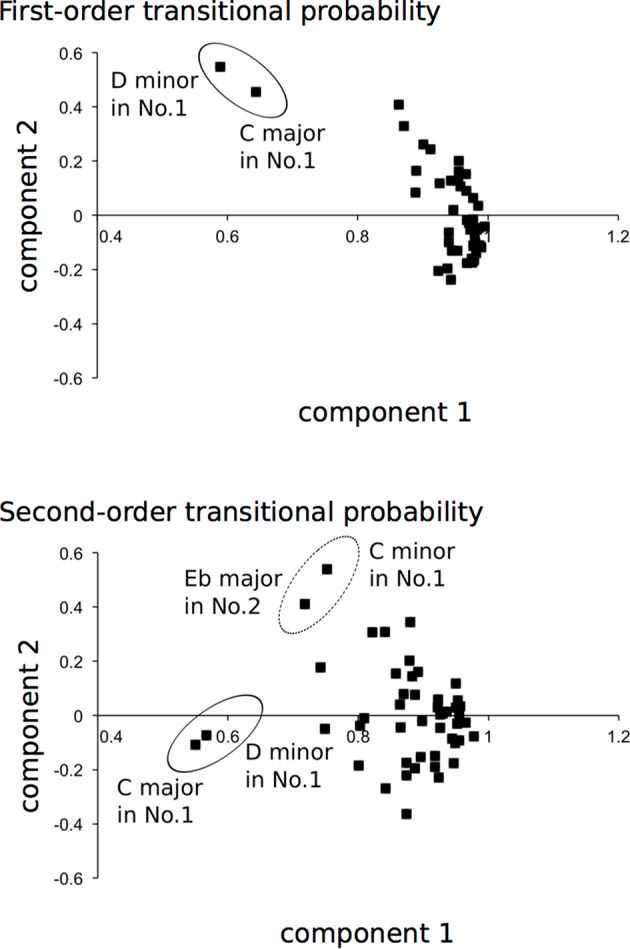
Principal component analysis scatter plots. The dots represent each piece of music in the Well-Tempered Clavier, which is a collection of two series (No. 1 and No. 2) in all 24 major and minor keys that was composed for solo keyboard by Johann Sebastian Bach. The dots in each circle represent pieces of music with the component of each related key: between D minor and C major, Eb major and C minor, and C major and D minor.

Based on the results of correlation analysis, the first- and second-order TPs, in which there are a number of correlations of at least 0.3 (Tabachnick and Fidell, 2007), were analyzed by principal component analysis. In the first-order TP, the decision was made to specify two principal component solutions (eigenvalue >1; **Table 2A** and Figure 5). The two principal components accounted for 92.4% of the total variance. All of the music loaded higher than 0.58 on component 1. The “*loadings*” can be understood as the weights for each original variable when calculating the principal component. Thus, the result explains the general component of the Well-Tempered Clavier. The C major and D minor in the first series (No. 1) of the Well-Tempered Clavier loaded higher than 0.45 on 2. This explains a component of related keys (i.e., the relative key of the subdominant key; Table 1) between C major and D minor. In the second-order TP, the decision was made to specify a three principal component solution (eigenvalue >1; Table 2B and Figure 5). The three principal components accounted for 83.2% of the total variance. All of the music loaded higher than 0.55 on 1,. This explains the general component of the Well-Tempered Clavier. On the other hand, compared to the other music, the C major and D minor in No. 1 of the Well-Tempered Clavier loaded <0.57 on component 1. The C minor in No. 1 and E♭ major in No. 2 of the Well-Tempered Clavier loaded at 0.41 or higher on component 2. This explains shared components of a related key (i.e., relative keys). The only D minor in No. 1 of the Well-Tempered Clavier loaded heavily (0.52) on component 3.

**Table 1:** Related key in all 24 major and minor keys.

**Major**	**Relative minor**	**Subdominant, dominant, and their relatives**	**Parallel minor**
C	Am	F, G, Dm, Em	Cm
G	Em	C, D, Am, Bm	Gm
D	Bm	G, A, Em, F#m	Dm
A	F#m	D, E, Bm, C#m	Am
E	C#m	A, B, F#m, G#m	Em
B	G#m	E, F#, C#m, D#m	Bm
G♭	E♭m	C♭, D♭, A♭m, B♭m	F#m
D♭	B♭m	G♭, A♭, E♭m, Fm	C#m
A♭	Fm	D♭, E♭, B♭m, Cm	G#m
E♭	Cm	A♭, B♭, Fm, Gm	E♭m
B♭	Gm	E♭, F, Cm, Dm	B♭m
F	Dm	B♭, C, Gm, Am	Fm

**Table 2A:** The results of principal component analysis.

	**1st-order transition probability**			**2nd-order transition probability**		
	**Total**	Variance ^*^	Cumulative ^*^	**Total**	Variance ^*^	Cumulative ^*^
Component 1	42.814	89.196	89.196	37.073	77.236	77.236
Component 2	1.548	3.225	92.421	1.528	3.184	80.419
Component 3				1.331	2.773	83.193

*The eigenvalue and percentages of variance and comulative variance*.

* *Percentage*.

**Table 2B:** The eigenvectors for the principal components.

		**1st-order transitonal probability**		**2nd-order transition probability**		
		**Component 1**	**Component 2**	**Component 1**	**Component 2**	**Component 3**
No. 1	C major	0.644	0.454	0.550	−0.109	−0.212
	C minor	0.968	−0.018	0.752	0.539	0.090
	Db major	0.912	0.243	0.809	−0.011	0.201
	Db minor	0.940	−0.063	0.918	−0.150	0.137
	D major	0.940	−0.100	0.864	0.040	−0.109
	D minor	0.589	0.547	0.567	−0.073	0.523
	Eb major	0.971	−0.030	0.947	−0.177	−0.017
	Eb minor	0.976	−0.175	0.952	−0.031	−0.200
	E major	0.946	−0.132	0.879	0.202	−0.289
	E minor	0.973	−0.053	0.926	−0.046	0.074
	F major	0.954	0.127	0.883	0.144	0.107
	F minor	0.990	−0.119	0.955	−0.093	−0.130
	Gb major	0.871	0.329	0.742	0.176	0.068
	Gb minor	0.980	−0.075	0.874	−0.222	−0.089
	G major	0.966	0.151	0.918	−0.190	0.173
	G minor	0.975	−0.160	0.953	0.055	−0.128
	Ab major	0.955	0.200	0.822	0.306	0.175
	Ab minor	0.953	−0.132	0.858	0.154	−0.173
	A major	0.943	0.128	0.874	−0.175	0.008
	A minor	0.988	−0.116	0.978	−0.077	−0.083
	Bb major	0.958	0.106	0.887	−0.195	0.051
	Bb minor	0.938	−0.197	0.926	0.003	−0.042
	B major	0.943	−0.238	0.922	0.029	−0.313
	B minor	0.968	−0.177	0.944	−0.086	−0.109
No. 2	C major	0.974	−0.051	0.880	0.344	−0.034
	C minor	0.977	−0.016	0.892	0.160	0.106
	Db major	0.901	0.261	0.865	−0.045	−0.026
	Db minor	0.983	−0.053	0.930	0.007	0.032
	D major	0.926	0.118	0.874	−0.364	0.171
	D minor	0.977	0.063	0.896	−0.154	0.063
	Eb major	0.955	0.162	0.718	0.411	0.342
	Eb minor	0.889	0.083	0.842	−0.270	0.251
	E major	0.987	−0.111	0.950	0.118	−0.011
	E minor	0.981	−0.103	0.924	−0.229	−0.167
	F major	0.979	−0.167	0.957	0.033	−0.169
	F minor	0.890	0.164	0.803	−0.038	0.214
	Gb major	0.967	0.090	0.887	0.076	0.026
	Gb minor	0.986	−0.047	0.923	0.059	0.046
	G major	0.863	0.408	0.749	−0.050	0.350
	G minor	0.947	0.020	0.870	0.079	−0.019
	Ab major	0.995	−0.042	0.965	−0.027	−0.050
	Ab minor	0.945	−0.130	0.898	−0.021	0.025
	A major	0.980	−0.110	0.841	0.307	−0.044
	A minor	0.924	−0.206	0.801	−0.186	−0.070
	Bb major	0.978	−0.113	0.950	0.028	−0.199
	Bb minor	0.982	−0.140	0.955	−0.001	−0.159
	B major	0.985	0.034	0.949	−0.102	0.052
	B minor	0.978	−0.114	0.936	0.013	−0.050

## Discussion

### Psychological Aspects of TP in Musical Sequence

Based on the information theory (Shannon, 1948) covering multi-order Markov models and the cognitive models, a tone with a higher TP may be one that a composer is more likely to choose than those with lower TPs. Thus, the TP distributions sampled from music may represent the musical characteristics based on a composer's statistical knowledge underlying prediction. The present study aimed to examine how the statistical structure interacts with tonality in music. To verify it in all 24 major and minor keys (Figure 1), the TPs of the sequences containing the highest pitches in Well-Tempered Clavier were calculated based on Markov stochastic models. It was hypothesized that the statistical structure in music interacts with tonality in music and that music-specific knowledge of tonality may modulates statistical knowledge in music.

### The Relationships Between Tonality and Hierarchy of Stochastic Structure in Music

The present study adopted the sequences that appear in all pieces of music (i.e., universal sequences in the Well-Tempered Clavier). The TP differences between major and minor keys could be detected in lower-order (1st and 2nd in Figure 3A) but not in higher-order hierarchical models. This implies that these sequences may have specific semantics in each major and minor key. In the context of statistical learning, the tonality may modulate a lower- rather than a higher-order statistical knowledge of music. The TPs in the fugue were higher than those in the prelude (Figure 3B), and the difference was prominent in sequences in which the interval was not more than a whole step (i.e., ±2), such as those found in musical scales (Figure 3F). It is well-known that the prelude less strictly follows the rules of Western classical music compared to the fugue. The findings in the present study may reflect the difference in statistical knowledge related to strategies for musical composition.

As a general tendency, the TPs of universal sequences were higher in minor than in major keys (Figures 3A,E). However, the difference became weaker in the series of No. 2 compared to that in No. 1 (Figure 3D). Statistical knowledge of universal sequences might be modulated from composition periods in No. 1 to No. 2. It would be interesting if the time-course variation of statistical structures may reflect the time-course variation of statistical knowledge. It is of note, however, that this study did not directly investigate the composer's statistical knowledge of music, as only the statistics of musical scores were analyzed. There may be other possible explanations for the findings of this study. For instance, it might have been Bach's intentional plan to compose music based on the statistical structure of music. Future studies should examine the effects of statistical knowledge on music compositions and neurological responses in parallel.

In the first- and second-order TPs, all of the pieces of music are related to each other (Supplementary Material and Figure 4). In the third- and fourth-order TPs, some of the music is related to each other, regardless of tonalities. There are more correlations in the third-order than fourth-order TPs. In the fifth- and sixth-order TPs, no remarkable correlations were detected. These results suggest that there are statistical characteristics that are shared among each piece of music at least in the first- and second-order hierarchical levels of statistical structure. In other words, there may be universal implicit knowledge of music in the composer at the lower hierarchical levels, regardless of tonalities and pitch frequencies. The higher the hierarchical levels of TPs, the less the music was correlated with each other. From information theoretical viewpoint, the statistical models at lower hierarchical levels increases joint probability and mutual information, whereas statistical structures at higher hierarchical levels are less correlated, and interpreted as surprisal information (Gupta and Bahmer, 2019). The combined increase in mutual information at lower hierarchical level and surprisal information at higher hierarchical level would serve as the basis of specific knowledge about music (Gupta and Bahmer, 2019). These results also suggest that the higher the hierarchical level of statistical structure, the stronger the independence of characteristics in each piece of music. The specific characteristics in each piece of music may exist in higher hierarchical levels of statistical structure. This may imply that greater creativity is attributed at higher hierarchical level (Daikoku, 2018b). Thus, it could be assumed that the general statistical structure that is shared among many pieces of music is formed by low-hierarchical implicit knowledge, whereas the specific structure that is independent of each piece of music is formed by high-hierarchical implicit knowledge (Gupta and Bahmer, 2019).

### J.S. Bach's Music for Study on Implicit and Explicit Knowledge

Johann Sebastian Bach (1685–1750), a German composer and musician of the Baroque period, is considered to have contributed to the development of musical tonality and has been central to Western classical music theory until the present (Rohrmeier and Cross, 2008). His music is often used to investigate the probabilities of musical sequences. Furthermore, to investigate the relationships between tonality and statistical structure in music, the Well-Tempered Clavier is considered an excellent medium because it is a collection of music containing all the keys of Western classical music (i.e., 24 major and minor keys). Thus, the statistical characteristics of each piece of music with a key in the Well-Tempered Clavier could be, in part, regarded as approximations of the statistical characteristics of the entire range of Western classical music in each key. In other words, the findings in the present study may reflect the implicit knowledge in each musical key in humans who explicitly learn the music-specific knowledge based on Western classical music and who intentionally follow these frameworks when composing music. Furthermore, the present study may suggest that there are at least two types of implicit knowledge that are dependent on and independent of tonality, respectively. This study, however, did not directly demonstrate that the implicit musical knowledge is reflected in music, as only the statistics of musical scores were analyzed. Future studies should investigate, in parallel, how implicit learning in music is reflected in the neurological response and how the learned knowledge is expressed when composing music.

The representative phrases of sequential patterns with mean highest and lowest probabilities were decoded as musical scores in Figure 2, based on each hierarchical level of first- (highest: *P*[−2|0], lowest: *P*[−16|0]), second- (highest: *P*[−3|0, −2], lowest: *P*[10|0, −1]), third- (highest: *P*[−5|0, −2, −4], lowest: *P*[−6|0, −4, −8]), fourth- (highest: *P*[−7|0, −2, −4, −5], lowest: *P*[0|0, 5, 1, −2]), fifth- (highest: *P*[−9|0, −2, −4, −5, −7], lowest: *P*[−2|0, −7, −8, −7, −3]), and sixth- (highest: *P*[−3|0, −1, −3, −5, −6, −5], lowest: *P*[−3|0, 8, 6, 7, 0, −2]). The sequential patterns with the highest sequential patterns are familiar ones in Western classical music, suggesting that implicit statistical knowledge and explicit music-specific knowledge interact, in part, with each other. The principal component analysis detected the shared components of related keys (Figure 5). This suggests that tonalities modulate implicit knowledge in music. However, these findings are not detected in all the types of related keys (Supplementary Material). Future studies will be needed to clarify the relationships between statistical structure and tonalities in music. In the present study, all of the pitch transitions were numbered to understand how the pitches, but not the notes, were transitioned to from the first pitch. This was performed to eliminate the effects of the change of key on sequential patterns. Thus, the results may represent statistics based on relative pitches rather than absolute pitches. Nonetheless, the present study suggests that explicit knowledge on tonality could, in part, modulate implicit knowledge in music.

## Conclusion

The present study indicated that, in the lower hierarchical levels of statistical structure (first and second orders), all the pieces of music are related to each other. However, the higher the hierarchical levels of TPs, the less the music was correlated with each other, regardless of tonality. These findings suggest that the general statistical structure that is shared among many pieces of music is formed by low-hierarchical implicit knowledge, whereas the specific structure that is independent of each piece of music is formed by high-hierarchical implicit knowledge. This may imply that greater creativity is attributed at higher hierarchical level. On the other hand, the principal component analysis detected the shared components of related keys, suggesting that tonalities modulate implicit knowledge in music. The implicit statistical knowledge and explicit music-specific knowledge could, in part, interact with each other. It is suggested that there are at least two types of implicit knowledge that are dependent on and independent of tonality, respectively. The present study sheds new light on novel methodologies that can be employed to evaluate the implicit knowledge of a composer using musical scores in interdisciplinary studies that include psychology, informatics, and musicology.

## Data Availability Statement

All datasets generated for this study are included in the manuscript/Supplementary Files.

## Author Contributions

The methodology of the present study was considered by the authors. The author analyzed all of the data and prepared the figures, and wrote the manuscript text.

### Conflict of Interest

The author declares that the research was conducted in the absence of any commercial or financial relationships that could be construed as a potential conflict of interest.

## Supplementary Material

Supplementary Table 1 The results of correlation analysis. The red, green, yellow cells represent strong (0.7 ≦ |r| < 1.0), moderate (0.4 ≦ |r| < 0.7), and weak (0.2 ≦ |r| < 0.4) correlations, respectively. M and m indicate major and minor keys, respectively.
